# Supplementary material for: Comparison of Long-Term Pneumonia Risk between Spleen Injury and Non-Spleen Injury after Total Splenectomy—A Population-Based Study
Source: J Pers Med. 2022 Feb 18;12(2):308. doi: 10.3390/jpm12020308 (PMC8877515; doi:10.3390/jpm12020308)
Supplement: Supplementary file 1 [file jpm-12-00308-s001.zip › jpm-1587986-supplementary.pdf]

Table S1. Risk factors for pneumonia according to spleen injury status, and the effect of spleen injury on the risk of pneumonia.

	Splenectomy due to Injury			Splenectomy not Due to an Injury			Ratio	Due to Spleen Injury *vs.* not Due to Spleen Injury (Reference)	
	Events	PYs	Rate (per 1000 PYs)	Events	PYs	Rate (per 1000 PYs)		Adjusted HR [1] (95% CI)	*p*
Overall	1917	123,167	15.56	720	60,004	12.00	1.297	1.567 (1.427–1.721)	<0.001
Sex									
Male	1303	67,402	19.33	567	43,053	13.17	1.468	1.776 (1.618–1.982)	<0.001
Female	614	55,766	11.01	153	16,951	9.03	1.220	1.472(1.341–1.615)	<0.001
Age group (years)									
20–44	192	18,771	10.23	210	22,769	9.22	1.109	1.340 (1.206–1.475)	<0.001
45–64	584	47,811	12.21	250	22,769	10.98	1.112	1.445 (1.389–1.526)	<0.001
≥65	1141	56,586	20.16	260	14,467	17.97	1.122	1.575 (1.431–1.798)	<0.001
Insurance premium (NT$)									
<18,000	1880	120,752	15.57	708	15,232	46.48	0.335	0.779 (0.586–1.120)	0.275
18,000–34,999	31	1811	17.12	12	1115	10.76	1.591	1.892 (1.534–2.010)	<0.001
≥35,000	6	604	9.93	0	43,657	0.00			0.896
Season									
Spring	479	26,793	17.88	209	14,491	14.42	1.240	1.497 (1.368–1.659)	<0.001
Summer	476	31,193	15.26	147	15,224	9.66	1.580	1.910 (1.732–2.101)	<0.001
Autumn	463	35,126	13.18	154	16,888	9.12	1.445	1.746 (1.584–1.916)	<0.001
Winter	499	30,056	16.60	210	13,401	15.67	1.060	1.281 (1.163–1.423)	<0.001
Urbanization level									
1 (highest)	643	43,846	14.66	161	13,966	11.53	1.272	1.537 (1.382–1.689)	<0.001
2	880	55,523	15.85	314	25,449	12.34	1.285	1.556 (1.420–1.713)	<0.001
3	98	7446	13.16	60	6161	9.74	1.351	1.634 (1.497–1.780)	<0.001
4 (lowest)	296	16,352	18.10	185	14,429	12.82	1.412	1.708 (1.526–1.897)	<0.001
Level of care									
Hospital center	811	57,652	14.07	202	16,821	12.01	1.171	1.402 (1.283–1.567)	<0.001
Regional hospital	771	50,948	15.13	321	29,913	10.73	1.410	1.703 (1.552–1.892)	<0.001
Local hospital	335	14,567	23.00	197	13,271	14.84	1.549	1.879 (1.700–2.101)	<0.001

[1] The adjusted HRs were calculated using Cox proportional hazards regression with all the variables listed in the table included in the model. CI, confidence interval; HR, hazard ratio; PYs, person-years.

Table S2. Factors of pneumonia with/without chest injury among spleen injury by using Cox regression.

Groups	Population	Events	Events%	Rate (per 1000 PYs)	Adjusted HR	*p*
Without spleen injury	5681	720	12.67	12.00	Reference	
With spleen injury	11,817	1917	16.22	15.56	1.567	<0.001
With spleen injury, without chest injry	11,665	1887	16.18	15.50	1.448	<0.001
With spleen injury, with chest injry	152	30	19.74	21.04	2.241	<0.001

Table S3. pneumonia with/without chest injury among spleen injury by using Cox regression.

Groups	Adjusted HR	*p*
With spleen injury, without chest injry	Reference	
With spleen injury, with chest injry	1.351	0.603
